# Survival outcomes in patients with chemotherapy-naive metastatic castration-resistant prostate cancer treated with enzalutamide or abiraterone acetate

**DOI:** 10.1038/s41391-021-00318-3

**Published:** 2021-02-21

**Authors:** Scott T. Tagawa, Krishnan Ramaswamy, Ahong Huang, Jack Mardekian, Neil M. Schultz, Li Wang, Rickard Sandin, Stanislav Lechpammer, Daniel J. George

**Affiliations:** 1 Weill Cornell Medicine, New York, NY USA; 2 Pfizer Inc., New York, NY USA; 3 STATinMED Research, Plano, TX USA; 4 Astellas Pharma Inc., Northbrook, IL USA; 5 Pfizer AB., Sollentuna, Sweden; 6 Pfizer Inc., San Francisco, CA USA; 7 Duke Cancer Institute, Duke University School of Medicine, Durham, NC USA

**Keywords:** Diseases, Prostate cancer

## Abstract

**Objective:**

Evaluation of the comparative effectiveness of enzalutamide and abiraterone in patients with metastatic castration-resistant prostate cancer (mCRPC) is limited to meta-analyses of randomized trials that exclude patients with significant comorbidities. We evaluated overall survival (OS) in patients with chemotherapy-naive mCRPC treated with enzalutamide or abiraterone acetate (abiraterone) in a real-world single payer setting.

**Methods:**

A retrospective analysis (4/1/2014–3/31/2018) of the Veterans Health Administration (VHA) database was conducted. Patients with mCRPC had ≥1 pharmacy claim for enzalutamide or abiraterone (first claim date = index date) following disease progression on surgical/medical castration, without chemotherapy <12 months prior to index date. Patients had continuous VHA enrollment for ≥12 months pre-index date and were followed until death, disenrollment, or end of study. Kaplan–Meier analysis and multivariable Cox proportional hazards regression models examined the OS treatment effect.

**Results:**

Patients with chemotherapy-naive mCRPC (*N* = 3174; enzalutamide, *n* = 1229; abiraterone, *n* = 1945) had mean ages of 74 and 73 years, respectively. Median follow-up was 18.27 and 19.07 months with enzalutamide and abiraterone, respectively. Enzalutamide-treated patients had longer median treatment duration than abiraterone-treated patients (9.93 vs 8.47 months, respectively, *p* = 0.0008). After baseline comorbidity adjustment, enzalutamide-treated patients had a 16% reduced risk of death (hazard ratio [HR] = 0.84; 95% CI, 0.76–0.94; *p* = 0.0012). For patients who remained on first line-therapy only, enzalutamide-treated patients had improved OS versus abiraterone-treated patients (HR = 0.71; 95% CI, 0.62–0.82). Enzalutamide-treated patients who crossed over to abiraterone had a comparable risk of death versus abiraterone-treated patients who crossed over to enzalutamide (HR = 1.10; 95% CI, 0.89–1.35). These results were confirmed by sensitivity analysis, which considered prognostic variables.

**Conclusions:**

Retrospective analysis of the VHA database indicated that chemotherapy-naive patients with mCRPC initiating therapy with enzalutamide had improved survival versus abiraterone.

## Introduction

Despite disease progression on androgen deprivation therapy, castration-resistant tumors usually remain dependent on androgen receptor (AR) signaling. This is evidenced by efficacy of AR pathway inhibitors to prolong survival, including enzalutamide, an androgen receptor antagonist, and abiraterone acetate, a specific inhibitor of CYP17 that blocks androgen synthesis administered with prednisone (hereafter referred to as abiraterone) [1–8].

The PREVAIL trial of chemotherapy-naive patients with metastatic castration-resistant prostate cancer (mCRPC) showed a median overall survival (OS) with enzalutamide of 35.5 months versus 31.3 months with placebo [4]. The reduced risk of death by 17% (hazard ratio [HR] = 0.83; 95% CI, 0.75–0.93; *p* < 0.0008) [4] with enzalutamide versus placebo was maintained despite subsequent therapies and crossover from placebo. In a similar population, COU-AA-302 showed an OS improvement with abiraterone versus placebo (median OS, 34.7 vs 30.3 months; HR, 0.81; 95% CI, 0.70–0.93; *p* < 0.0033) despite crossover from placebo and subsequent therapies in both study arms [8].

Several indirect analyses have compared OS improvement in chemotherapy-naive patients with mCRPC treated with enzalutamide versus abiraterone in lieu of direct prospective comparisons. Two independent meta-analyses of clinical trials that included PREVAIL and COU-AA-302 showed evidence of better OS with enzalutamide versus abiraterone [9, 10], while one network meta-analysis did not show a survival difference [11]. All three studies showed a statistically significant improvement in progression-free survival with enzalutamide versus abiraterone [9–11].

Generalizations from the controlled setting of randomized clinical trials may have limitations because they often exclude patients with significant comorbidities. To date, few studies evaluated the comparative effectiveness of abiraterone and enzalutamide on OS in the real-world setting. The Veterans Health Administration (VHA) database is the largest integrated healthcare system in the United States (US), and therefore serves as a good index of real-world prostate cancer experience in this country, particularly since prostate cancer is the most common cancer diagnosis among US veterans [12].

In the current study, we investigated the comparative effectiveness of the novel hormonal therapies (NHTs) enzalutamide and abiraterone on OS in chemotherapy-naive patients with mCRPC in a US veterans’ population treated in the VHA health system.

## Methods

### Data source

This was a retrospective observational study of the VHA database from April 1, 2013 to March 31, 2018. The database includes: inpatient, outpatient, cost, laboratory, pharmacy, and vital status files. Medical claims comprised diagnosis and procedure codes from the International Classification of Diseases, Ninth and Tenth Revisions, Clinical Modification (ICD-9-CM & ICD-10-CM), Current Procedural Terminology, and the Healthcare Common Procedure Coding System. Outpatient pharmacy claims were per the National Drug Codes for dispensed medications. This study was exempt from institutional review board assessment.

### Patient identification

**Fig. 1:**
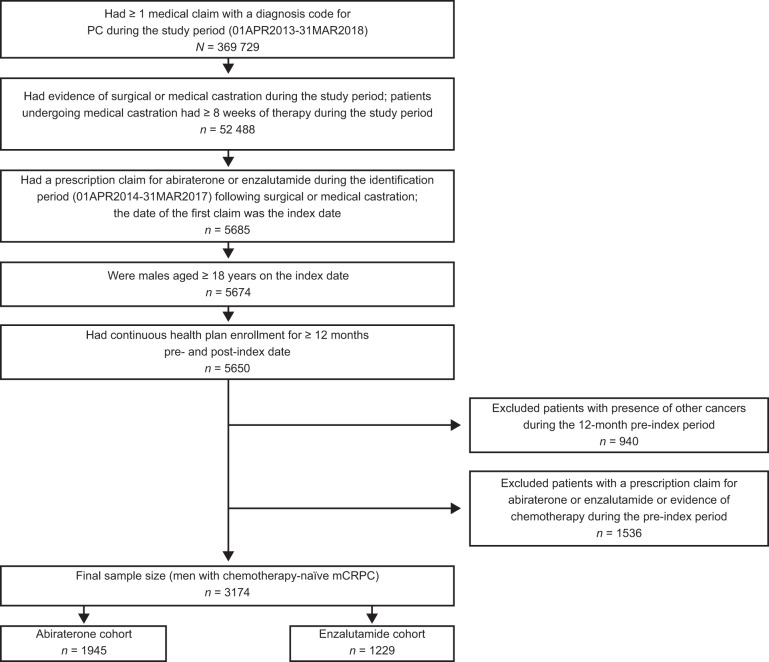
Patient attrition. mCRPC metastatic castration-resistant prostate cancer.

Eligibility criteria included adult patients (aged ≥ 18 years), ≥1 medical claim with a prostate cancer diagnosis code (ICD-9-CM: 185, ICD-10-CM: C61), evidence of surgical or medical castration, and a post-castration prescription claim for enzalutamide or abiraterone at any time during the study (see Fig. 1 for full details). Patients were assigned to distinct cohorts based on index prescription: enzalutamide or abiraterone. Discontinuation was defined as a treatment gap of ≥90 days post-index prescription.

### Patient characteristics

Demographic characteristics were examined on index date. Clinical characteristics were measured during the baseline period included modified Quan-Charlson comorbidity index (CCI) scores, individual comorbidities, prior prostate cancer treatments, and prognostic variables (Table 1).

**Table 1:** Demographic and baseline characteristics of patients with chemotherapy-naive mCRPC treated with enzalutamide or abiraterone.

Demographic and baseline clinical characteristics	Enzalutamide (*n* = 1229)	Abiraterone (*n* = 1945)	*p* value	SMD
*Mean age, years (SD)*	73.97 (7.68)	73.18 (7.88)	**0.0112**	10.05
18–64	99 (8.06)	202 (10.39)	**0.0291**	8.06
65–74	469 (38.16)	755 (38.82)	0.7113	1.35
75–88	476 (38.73)	709 (36.45)	0.1962	4.70
> 89	185 (15.05)	279 (14.34)	0.5821	2.00
*Race, no. (%)*				
White	801 (65.17)	1322 (67.97)	0.1032	5.92
Black	317 (25.79)	470 (24.16)	0.3006	3.76
Other	33 (2.69)	35 (1.80)	0.0932	5.98
Unknown	78 (6.35)	118 (6.07)	0.7497	1.16
*Quan-CCI Score (SD)*	6.37 (3.57)	6.42 (3.49)	0.6613	1.59
*Individual comorbidities, no. (%)*				
Urinary tract infection	151 (12.29)	202 (10.39)	0.0971	6.00
Impotence	88 (7.16)	170 (8.74)	0.1126	5.84
Hypertension	870 (70.79)	1347 (69.25)	0.3587	3.35
Stroke	65 (5.29)	136 (6.99)	0.0549	7.10
Angina pectoris perforation	28 (2.28)	38 (1.95)	0.5325	2.25
Arrhythmia	109 (8.87)	109 (5.60)	**0.0004**	12.62
Congestive heart failure	121 (9.85)	125 (6.43)	**0.0005**	12.52
Hyperlipidemia	664 (54.03)	1036 (53.26)	0.6746	1.53
Type 2 diabetes	432 (35.15)	575 (29.56)	**0.001**	11.96
Liver damage/abnormality	75 (6.1)	114 (5.86)	0.7796	1.02
Acute coronary syndrome/myocardial infarction	35 (2.85)	61 (3.14)	0.644	1.69
*Pre-index treatments, no. (%)*				
Radiation therapy	48 (3.91)	85 (4.37)	0.5246	2.33
Steroid therapy (chronic corticosteroid use)	113 (9.19)	157 (8.07)	0.2695	4.00
*Baseline characteristics included in sensitivity analysis, mean (SD)*				
Mean PSA, ng/mL	111.58 (286.89)	128.65 (384.62)	0.1671	5.03
Mean alkaline phosphatase, U/L	153.95 (214.45)	158.14 (238.15)	0.6238	1.85
Mean hemoglobin, g/dL	12.21 (1.80)	12.28 (1.84)	0.2969	4.07

Any category with less than 11 patients cannot be reported due to HIPPA regulations. Bold values indicate statistically significant differences between cohorts. SMDs > 10 indicate practically/clinically significant differences between cohorts. All the characteristics shown in the above table were included in the Cox proportional model analysis for the estimation of overall survival.

*CCI* Charlson comorbidity index, *HIPPA* Health Insurance Portability and Accountability Act, *mCRPC* metastatic castration-resistant prostate cancer, *PSA* prostate specific antigen, *SD* standard deviation, *SMD* standardized mean difference.

### Outcome measures

Survival was defined as the duration from index date to death. Patients without a defined date of death at the end of study were assumed alive and censored. First, OS was compared between the enzalutamide and abiraterone cohorts in the overall population (i.e., full study population regardless of sequential treatment). Second, OS was compared among patients who received first-line treatment with enzalutamide without any sequential treatment (enzalutamide only) or first-line abiraterone without any sequential treatment (abiraterone only). Third, OS was evaluated among patients switching therapies from enzalutamide to abiraterone versus switching from abiraterone to enzalutamide. Fourth, OS was evaluated in patients who switched from first-line treatment with enzalutamide or abiraterone to second-line chemotherapy. The third and fourth OS analyses included only patients who did not receive additional lines of therapy beyond the second line. Lastly, OS was evaluated in patients subsequently treated with other subsequent therapy termed “enzalutamide other” or “abiraterone other” who were patients receiving more than two lines of therapy. Subsequent therapies included enzalutamide, abiraterone, radium-223, sipuleucel-T, or chemotherapy (i.e., docetaxel, cabazitaxel, mitoxantrone) in various permutations and combinations.

### Statistical analysis

Descriptive statistics were conducted on all study variables. Counts and percentages (%) were reported for categorical variables; means and standard deviation were reported for continuous variables. Between-treatment comparisons were conducted using chi-square tests (for categorical variables) and 2-sample *t*-tests (for continuous variables). Kaplan–Meier analysis and a multivariable Cox proportional hazards regression model adjusting for the available baseline characteristics: age, race, CCI, individual comorbidities, and pre-index treatments were used to assess treatment-survival association and calculate HRs of death with 95% CIs. Sensitivity analysis was conducted including known mCRPC prognostic variables recorded in the VHA database: prostate specific antigen (PSA), hemoglobin, and alkaline phosphatase as covariates within 6 months prior to the index date [13–15]. Missing values of these factors were imputed using the *k*-Nearest-Neighbor technique [16, 17].

## Results

Patients with mCRPC (*N* = 5685) were identified using an enzalutamide or abiraterone prescription as a proxy indicator. After applying selection criteria, the total sample size comprised 3174 patients: (1229 patients in the enzalutamide cohort and 1945 patients in the abiraterone cohort) (Fig. 1).

### Baseline characteristics

Baseline demographics and clinical characteristics were generally similar; however, patients in the enzalutamide cohort were older and had a greater likelihood for cardiac arrhythmia, congestive heart failure, and type 2 diabetes (Table 1).

### Treatment patterns in the enzalutamide and abiraterone cohorts

Treatment patterns were generally similar across cohorts (Supplementary Table 1). Approximately half of patients in the respective cohorts received one line of treatment only (enzalutamide, *n* = 668 [54%]; abiraterone, *n* = 889 [46%]). These included patients who were still on first-line treatment at the point of data cut (enzalutamide, *n* = 333 [27%], abiraterone *n* = 376 [19%]) and patients who stopped treatment without receiving any other active treatment (enzalutamide, *n* = 335 [27%], abiraterone *n* = 513 [26%]). Approximately one-quarter of patients crossed over from their prescribed first-line NHT to receive the alternative NHT only; 23% (*n* = 282) of patients crossed over from enzalutamide to abiraterone, and 26% (*n* = 504) crossed over from abiraterone to enzalutamide. An additional 6% of enzalutamide patients (*n* = 77) and 9% of abiraterone patients (*n* = 178) switched to chemotherapy as second-line treatment only. First-line enzalutamide or abiraterone followed by “other” multiple lines of treatment was observed in 20% (*n* = 241) and 25% (*n* = 478) of patients, respectively (Supplementary Table 2). This included patients who crossed over from one NHT to the other and then went on to receive other treatment (enzalutamide, *n* = 78 [6%]; abiraterone, *n* = 191, [10%]). Subsequent radium-223 or sipuleucel-T was observed in ≤1% of patients in either cohort.

### Pairwise comparison in the overall population

**Fig. 2:**
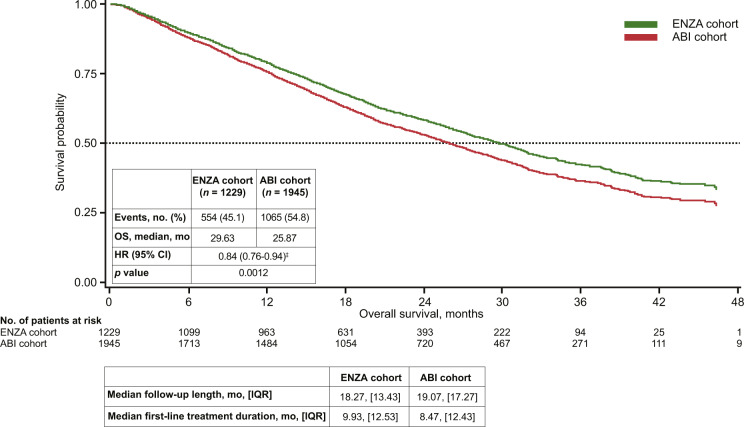
Adjusted Kaplan–Meier curve for overall survival in all patients with chemotherapy-naive mCRPC treated with either enzalutamide or abiraterone irrespective of follow-up treatments. *Enzalutamide versus abiraterone. ABI abiraterone, ENZA enzalutamide, HR hazard ratio, IQR interquartile range, mCRPC metastatic castration-resistant prostate cancer, OS overall survival.

Overall, median follow-up was similar: 18.27 months in the enzalutamide cohort (*n* = 1229) and 19.07 months in the abiraterone cohort (*n* = 1945). Median enzalutamide treatment duration was 9.93 months versus 8.47 months with abiraterone. After adjusting for age, race, individual comorbidities, and pre-index treatments, enzalutamide-treated patients had a 16% lower risk of death versus abiraterone-treated patients (adjusted HR = 0.84; 95% CI, 0.76–0.94; *p* = 0.0012) (Fig. 2). Median OS was longer in the enzalutamide cohort (29.63 months) versus the abiraterone cohort (25.87 months).

### Pairwise comparison with patients receiving first-line treatment only

**Fig. 3:**
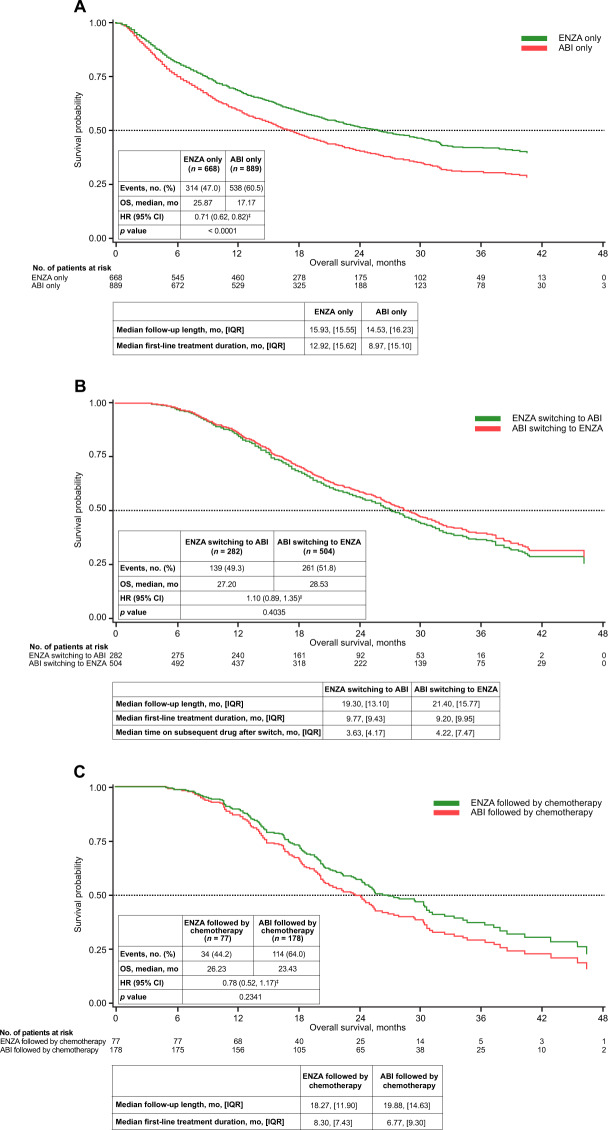
Adjusted Kaplan-Meier curves for overall survival by treatment subsets based on subsequent therapy. Adjusted Kaplan–Meier curve for overall survival **A** in patients who remained on enzalutamide only or abiraterone only*; **B** after crossover, switching from enzalutamide to abiraterone vs switching from abiraterone to enzalutamide; and **C** in patients who had enzalutamide followed by chemotherapy^†^ vs abiraterone followed by chemotherapy. *Patients were either persistently on ABI or ENZA and censored at month 48 or discontinued treatment without a second-line treatment. ^†^Chemotherapy included docetaxel, cabazitaxel, and mitoxantrone hydrochloride. ^‡^Enzalutamide versus abiraterone. ABI abiraterone, ENZA enzalutamide, HR hazard ratio, IQR interquartile range, OS overall survival.

Median follow-up among patients receiving first-line treatment only was 15.93 months in enzalutamide-treated patients and 14.53 months with abiraterone. Median first-line treatment duration was 12.92 months for enzalutamide-treated patients versus 8.97 months for abiraterone-treated patients. After baseline covariate adjustment, enzalutamide-treated patients had a 29% lower risk of mortality versus abiraterone-treated patients (adjusted HR = 0.71; 95% CI, 0.62–0.82; referenced to abiraterone-only patients) (Fig. 3A). Median OS was 25.87 months for enzalutamide-only patients versus 17.17 months for abiraterone-only patients.

### Pairwise comparison with crossover to NHT only

Median follow-up among patients who crossed over was 19.30 months in the enzalutamide subset and 21.40 months in the abiraterone subset. Median treatment duration was similar in enzalutamide patients who switched to abiraterone versus abiraterone patients who switched to enzalutamide (first-line: 9.77 vs 9.20 months; duration on subsequent drug after crossover: 3.63 vs 4.22 months). After baseline covariate adjustment, OS was comparable across subsets with an adjusted HR of 1.10 (95% CI, 0.89–1.35, referenced to abiraterone to enzalutamide crossover) with comparable median OS of 27.20 months in the enzalutamide to abiraterone subset versus 28.53 months in the abiraterone to enzalutamide subset (Fig. 3B).

### Pairwise comparison with switching to second-line chemotherapy only

Median follow-up in the enzalutamide subset for patients who switched to chemotherapy only was 18.27 months and 19.88 months in the abiraterone subset. Median duration of first-line treatment with enzalutamide was 8.30 months versus 6.77 months with abiraterone. Patients switching to chemotherapy demonstrated a trend to longer OS with first-line enzalutamide versus first-line abiraterone (adjusted HR = 0.78; 95% CI, 0.52–1.17; referenced from abiraterone to chemotherapy). Median OS was 26.23 months in enzalutamide-treated patients followed by chemotherapy versus 23.43 months in abiraterone-treated patients followed by chemotherapy (Fig. 3C).

### Pairwise comparison with switching to other sequential regimens

Median follow-up in the enzalutamide subset for patients who switched from enzalutamide to multiple other sequential therapies (Supplementary Table 2) was 24.03 months and 29.00 months in the abiraterone subset. Pairwise comparison of patients switching to other sequential therapies demonstrated similar OS in the enzalutamide subset versus the abiraterone subset (HR = 1.07; 95% CI, 0.79–1.43; referenced to abiraterone other) (Supplementary Fig. 1).

### Sensitivity analysis

**Fig. 4:**
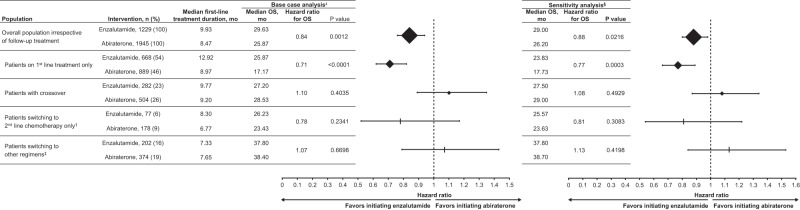
Overall survival and treatment duration in patients with chemotherapy-naive mCRPC treated with either enzalutamide or abiraterone in overall population and subsets based on subsequent therapy. *Adjusted for the following covariates: age, race, modified Quan-Charlson comorbidity index, individual comorbidities (urinary tract infection, impotence, hypertension, stroke, angina pectoris perforation, arrhythmia, congestive heart failure, hyperlipidemia, type 2 diabetes, liver damage/abnormality, and acute coronary syndrome/myocardial infarction), and pre-index treatments. ^†^Chemotherapy included docetaxel, cabazitaxel, and mitoxantrone hydrochloride. ^‡^Other = any sequential treatment sequence (excluding treatment sequences as defined in the above rows). ^§^Adjusted for the covariates included in the base case analysis as well as prostate specific antigen, hemoglobin, and alkaline phosphatase within 6 months prior to the index date. mCRPC metastatic castration-resistant prostate cancer.

Sensitivity analysis that further adjusted for baseline PSA, hemoglobin, and alkaline phosphatase confirmed our base case findings of a statistically significant OS effect in favor of enzalutamide over abiraterone in the overall population and in the subset receiving first-line treatment only, and no significant difference in the other subsets (Fig. 4).

## Discussion

In this retrospective cohort study using recent real-world data from the VHA, chemotherapy-naive patients with mCRPC, patients who initiated treatment with enzalutamide versus abiraterone had a longer median treatment duration and a 16% reduced risk of death. Similarly, in the subset receiving first-line NHT only (≈50% of the overall population), a 29% reduced risk of death was shown for enzalutamide versus abiraterone. In contrast, in subset analyses of patients who crossed over from one NHT to the other, median OS was comparable. Similarly, no statistically significant difference in OS was observed between enzalutamide or abiraterone after switching to chemotherapy. Our primary finding of improved OS with enzalutamide versus abiraterone is supported by meta-analyses that have either shown a greater survival advantage for enzalutamide [10] or a trend toward survival advantage for enzalutamide over abiraterone [9, 11]. Meta-analyses have consistently shown a longer progression-free survival for enzalutamide compared with abiraterone [9–11]. Our findings are also consistent with real-world data comparing treatment duration of the respective agents. Utilizing commercial claims data, Shultz et al. demonstrated that enzalutamide-treated patients with chemotherapy-naive mCRPC remained on treatment longer than abiraterone-treated patients (10.7 months vs 8.8 months; *p* = 0.008) [18].

To our knowledge, this is the first real-world study to evaluate the comparative effectiveness of abiraterone and enzalutamide on OS. A major strength of our study is the utilization of the VHA database which represents the largest integrated healthcare system in the United States, with prostate cancer being the most common cancer diagnosis [12]. In 2014, the incidence of newly diagnosed prostate cancer in the VHA database was 12,000, and the prevalence rate of survivorship was 200,000 [19]. As such, this patient population constitutes a large proportion of prostate cancer patients in the United States. Furthermore, this population has access to approved drugs without biases based on insurance preferences or “out-of-pocket” copayments.

In the current study, we have limited insights into the rationale for initial or subsequent therapeutic selections. Unlike the overall population and the subset receiving first-line NHT only, no survival advantage was conferred in the subset of patients (*n* = 786; one-quarter of the overall population) who crossed over to their respective alternative NHT only. In a prospective, multicenter phase 2 open-label crossover study of 202 patients with mCRPC, time to second PSA progression was longer for abiraterone followed by enzalutamide versus enzalutamide followed by abiraterone. Nevertheless, OS results were not statistically significantly different despite a majority of patients crossing over per protocol [20]. Therefore, our study supports this evidence of no notable OS differences favoring directional crossover from one NHT to the other.

In our study, evidence that the subset of patients who crossed over from one NHT to another had different characteristics from those who were treated with only one NHT may be inferred from the markedly longer median treatment duration on enzalutamide than abiraterone among those who only received first-line NHT only (12.92 vs 8.97 months) and the similarity in median treatment durations among patients who crossed over to the alternative NHT (9.77 with enzalutamide vs 9.20 months with abiraterone). We hypothesize that the NHT to NHT subsets may be enriched for those patients who developed subtle, subclinical progression (PSA increase with low volume radiographic changes or no changes); whereas those in the enzalutamide or abiraterone alone cohort may include those with more rapid mCRPC progression.

While there was a trend to better OS for enzalutamide-treated patients with second-line treatment with chemotherapy, this group was relatively underrepresented in our data (*n* = 255 patients). The overall population may have been enriched for patients who are not chemotherapy candidates, as evidenced by the relatively small proportion of patients (8%) who actually received second-line chemotherapy in this population. However, these rates of chemotherapy are consistent with previous real-world research [21].

We did not see a difference in OS in patients who switched from enzalutamide or abiraterone to other multi-line regimens (aside from direct crossover only, first-line treatment only, or second-line treatment with chemotherapy only). These patients may have a more indolent progression of cancer, but because each particular treatment pattern here is underrepresented, it was not feasible to trace their relative effectiveness.

Nearly half (49.05%) of the VHA patients analyzed received one line of treatment only, which may signal undertreatment in the VHA population. However, due to a median follow-up time of less than 20 months, 50% of these patients were still on treatment and some are therefore expected to receive additional therapies. Our findings are in line with real-world results from the Flatiron Health database wherein 51% of patients received only one line of therapy [21]. Similarly, the share of patients receiving sequential NHTs were at least as high in the VHA compared with the Flatiron Health database (17–25%) [21]. However, some degree of therapeutic inertia cannot be ruled out in the VHA as there was evidence of a lower use of radium-223 dichloride and sipuleucel-T in the VHA database (≤1%) relative to the Flatiron Health database (2–8%) [21].

Limitations of our study included those inherent to studies utilizing administrative claims databases, including possible coding errors or incorrect diagnoses entered. Our results may not be generalizable to younger patients, as VHA enrollees are predominantly older. Results of the VHA database analysis may not be generalizable to US populations of PC patients with other insurance. Importantly, the reported survival outcomes may be affected by differences with respect to disease severity, performance status, and other baseline patient characteristics that were not captured. Key prognostic variables such Gleason score, Eastern Cooperative Oncology Group performance status score, and number of metastases could not be controlled for. The direction of a potential bias for not being able to include these variables is unknown.

In conclusion, these results support the hypothesis that treatment with enzalutamide may lead to a reduced risk of death and prolonged survival versus abiraterone in patients with chemotherapy-naive mCRPC. However, the above limitations should be considered when applying these results. As both enzalutamide and abiraterone are likely to be used more frequently in earlier disease settings, including hormone-sensitive metastatic prostate cancer, it is important to know if there are real-world efficacy differences among these agents. These results support the rationale for prospective comparative effectiveness studies in the future.

## Supplementary information

Supplement

Supplemental Figure 1
