# Correction: Survival outcomes in patients with chemotherapy-naive metastatic castration-resistant prostate cancer treated with enzalutamide or abiraterone acetate

**DOI:** 10.1038/s41391-022-00578-7

**Published:** 2022-07-15

**Authors:** Scott T. Tagawa, Krishnan Ramaswamy, Ahong Huang, Jack Mardekian, Neil M. Schultz, Li Wang, Rickard Sandin, Stanislav Lechpammer, Daniel J. George

**Affiliations:** 1 Weill Cornell Medicine, New York, NY USA; 2 Pfizer Inc., New York, NY USA; 3 STATinMED Research, Plano, TX USA; 4 Astellas Pharma Inc., Northbrook, IL USA; 5 Pfizer AB., Sollentuna, Sweden; 6 Pfizer Inc., San Francisco, CA USA; 7 Duke Cancer Institute, Duke University School of Medicine, Durham, NC USA

Correction to: *Prostate Cancer and Prostatic Diseases* 10.1038/s41391-021-00318-3, published online 21 February 2021

In the sentence beginning ‘After adjusting for age...’ in this article, the value of upper CI limit ‘0.84’ should have read ‘0.94’.

In the sentence beginning ‘Unlike the overall population...’ in this article, the value ‘*n* = 700’ should have read ‘*n* = 786’.

In the sentence beginning ‘Nearly half (46,5%)...’ in this article, the value ‘46,5%’ should have read ‘49,5%’.

In Table 2 in the Supplementary PDF for this article, the line ‘Abiraterone followed by chemotherapy (c) 74 (3.80)’ has been deleted.

The original article has been corrected.

