# Supplementary material for: Survival outcomes in patients with chemotherapy-naive metastatic castration-resistant prostate cancer treated with enzalutamide or abiraterone acetate
Source: Prostate Cancer Prostatic Dis. 2021 Feb 21;24(4):1032–40. doi: 10.1038/s41391-021-00318-3 (PMC8616757; doi:10.1038/s41391-021-00318-3)
Supplement: Supplementary file 1 — Supplement [file 41391_2021_318_MOESM1_ESM.docx]

**Supplement**

**Supplementary Table 1.** Treatment sequence among patients with chemotherapy-naïve mCRPC prescribed abiraterone vs enzalutamide

| **Enzalutamide**  **(n = 1229)** | | **Abiraterone**  **(n = 1945)** | |
| --- | --- | --- | --- |
| **Treatment sequences** | **no. (%)** | **Treatment sequences** | **no. (%)** |
| Enzalutamide irrespective of follow-up treatments | 1229 (100) | Abiraterone irrespective of follow-up treatments | 1945 (100) |
| Enzalutamide followed by abiraterone | 282 (23) | Abiraterone followed by enzalutamide | 504 (26) |
| Enzalutamide followed by chemotherapy* as second-line therapy | 77 (6) | Abiraterone followed by chemotherapy* as second-line therapy | 178 (9) |
| Enzalutamide only (one line of therapy) | 668 (54) | Abiraterone only (one line of therapy) | 889 (46) |
| Enzalutamide followed by other^†^ | 202 (16) | Abiraterone followed by other^†^ | 374 (19) |

*Chemotherapy included docetaxel, cabazitaxel, and mitoxantrone hydrochloride. ^†^Other = any sequential treatment sequence (excluding treatment sequences as defined in the above rows).

*mCRPC* metastatic castration-resistant prostate cancer.

**Supplementary Table 2.** Treatment sequence among patients with chemotherapy-naïve mCRPC prescribed abiraterone vs enzalutamide followed by “other” regimens (ie, aside from one line of therapy only, direct crossover to the other NHT only, or chemotherapy as second-line therapy)

| **Enzalutamide**  **(n = 1229)** | | **Abiraterone**  **(n = 1945)** | | |
| --- | --- | --- | --- | --- |
| **Treatment sequences** | **no. (%)** | **Treatment sequences** | **no. (%)** | |
| Enzalutamide followed by enzalutamide (e)* | 69 (5.61) | Abiraterone followed by enzalutamide (e), followed by chemotherapy (c) | | 134 (6.89) |
| Enzalutamide followed by abiraterone (a), followed by chemotherapy (c) | 62 (5.04) | Abiraterone followed by abiraterone (a)* | | 84 (4.32) |
| Enzalutamide followed by c, followed by a | 21 (1.71) | Abiraterone followed by c, followed by e | | 55 (2.83) |
| Enzalutamide followed by a, followed by e | 16 (1.30) | Abiraterone followed by a, followed by e* | | 27 (1.39) |
| Enzalutamide followed by e, followed by a | 13 (1.06) | Abiraterone followed by e, followed by e | | 25 (1.29) |
| Enzalutamide followed by e, followed by e* | 12 (0.98) | Abiraterone followed by e, followed by a | | 22 (1.13) |
| Other regimens^†^ | 48 (3.91) | Abiraterone followed by c, followed by e, followed by c | | 18 (0.93) |
|  |  | Abiraterone followed by e, followed by c, followed by a | | 10 (0.51) |
|  |  | Other regimens^†^ | | 103 (5.30) |

*A 90-day gap in prescription dispensing was used to identify treatment discontinuation. Thus patients who had such a discontinuation and restarted therapy even with the same medication are shown here.

^†^Sum of other regimens with <10 patients per sequence.

*a* abiraterone; *e* enzalutamide; *c* chemotherapy; *NHT* novel hormonal therapy.

**Supplemental Fig. 1**. Adjusted Kaplan-Meier curve for overall survival, enzalutamide followed by other vs abiraterone followed by other


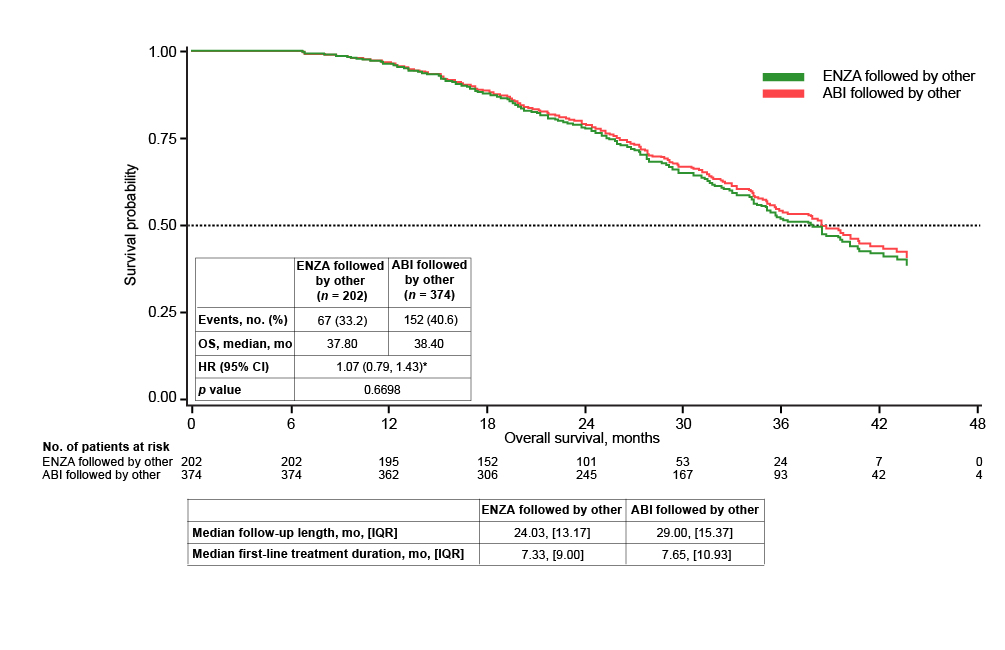


*Enzalutamide versus abiraterone.

*ABI* abiraterone; *ENZA* enzalutamide; *HR* hazard ratio; *IQR* interquartile range; OS overall survival.
